# NeuroBlu: a natural language processing (NLP) electronic health record (EHR) data analytic tool to generate real-world evidence in mental healthcare

**DOI:** 10.1192/j.eurpsy.2022.286

**Published:** 2022-09-01

**Authors:** R. Patel, S.N. Wee, R. Ramaswamy, S. Thadani, G. Guruswamy, R. Garg, N. Calvanese, M. Valko, A. Rush, M. Rentería, J. Sarkar, S. Kollins

**Affiliations:** 1 King’s College London, Academic Psychiatry, London, United Kingdom; 2 Holmusk, Usa, New York, United States of America; 3 Curbstone Consultant, Llc, Santa Fe, United States of America

**Keywords:** RWE, NLP, EHR, RWD

## Abstract

**Introduction:**

EHRs contain a rich source of real-world data that can support evidence generation to better understand mental disorders and improve treatment outcomes. However, EHR datasets are complex and include unstructured free text data that are time consuming to manually review and analyse. We present NeuroBlu, a secure, cloud-based analytic tool that includes bespoke NLP software to enable users to analyse large volumes of EHR data to generate real-world evidence in mental healthcare.

**Figure:**
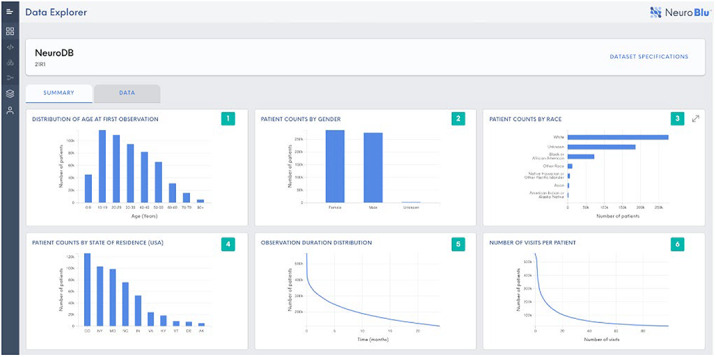

**Objectives:**

(i) To assemble a large mental health EHR dataset in a secure, cloud-based environment.

(ii) To apply NLP software to extract data on clinical features as part of the Mental State Examination (MSE).

(iii) To analyse the distribution of NLP-derived MSE features by psychiatric diagnosis.

**Methods:**

EHR data from 25 U.S. mental healthcare providers were de-identified and transformed into a common data model. NLP models were developed to extract 241 MSE features using a deep learning, long short-term memory (LSTM) approach. The NeuroBlu tool (https://www.neuroblu.ai/) was used to analyse the associations of MSE features in 543,849 patients.

**Figure:**
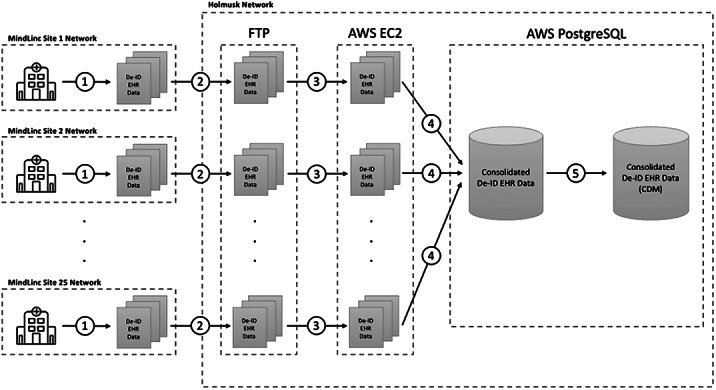

**Results:**

The figure below illustrates the percentage of patients in each diagnostic category with at least one recorded MSE feature.

**Figure:**
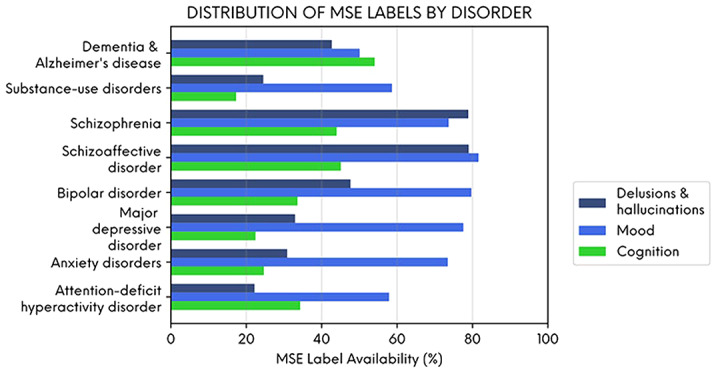

**Conclusions:**

Delusions and hallucinations were more likely to be recorded in people with schizophrenia and schizoaffective disorder, and cognitive features were more likely to be recorded in people with dementia. However, mood symptoms were frequently recorded across all diagnoses illustrating their importance as a transdiagnostic clinical feature. NLP-derived clinical information could enhance the potential of EHR data to generate real-world evidence in mental healthcare.

**Disclosure:**

This study was funded in full by Holmusk.

